# Pancreatic Ductal Organoids React Kras Dependent to the Removal of Tumor Suppressive Roadblocks

**DOI:** 10.1155/2019/2079742

**Published:** 2019-05-19

**Authors:** Lukas Perkhofer, Melanie Engler, Johann Gout, Frank Arnold, Mareen Morawe, Markus Breunig, Thomas Seufferlein, Alexander Kleger, Pierre-Olivier Frappart

**Affiliations:** Department of Internal Medicine I, University Hospital Ulm, Albert Einstein Allee 23, 89081 Ulm, Germany

## Abstract

Pancreatic ductal adenocarcinoma (PDAC) is still the Achilles heel in modern oncology, with an increasing incidence accompanied by a persisting high mortality. The developmental process of PDAC is thought to be stepwise via precursor lesions and sequential accumulation of mutations. Thereby, current sequencing studies recapitulate this genetic heterogeneity in PDAC and show besides a handful of driver mutations (KRAS, TP53) a plethora of passenger mutations that allow to define subtypes. However, modeling the mutations of interest and their effects is still challenging. Interestingly, organoids have the potential to recapitulate *in vitro*, the *in vivo* characteristics of the tissue they originate from. Here, we could establish and develop tools allowing us to isolate, culture, and genetically modify ductal mouse organoids. Transferred to known effectors in the IPMN-PDAC sequence, we could reveal significantly increased proliferative and self-renewal capacities for PTEN and RNF43 deficiency in the context of oncogenic KRAS^G12D^ in mouse pancreatic organoids. Overall, we were able to obtain promising data centering ductal organoids in the focus of future PDAC research.

## 1. Introduction

Independent of the rapidly progressing field of oncology that steadily overwhelms us with novel therapies in several entities, pancreatic ductal adenocarcinoma (PDAC) seems to be unaffected. Overall survival times only slightly improved over the last decades and will rank PDAC as the second most cause of cancer deaths within the next decade [1]. Reasons for this trend are manifold starting with epidemiologic and diagnostic and ending with biologic specificities [2, 3]. By now, we know three different types of PDAC precursor lesions: pancreatic intraepithelial neoplasia (PanIN), mucinous cystic neoplasm (MCN), and intraductal papillary mucinous neoplasm (IPMN), each following separate genetic routes towards PDAC [4]. The diverse mutational spectrum with few key driver mutations (e.g., *KRAS*, *TP53*, and *CDKN2A*) joined by a high number of passenger mutations causes the characteristic intra- and intertumoral heterogeneity of each PDAC [5, 6]. Specifically, oncogenic *KRAS* is the ultimate driving force of the PanIN-PDAC program, while the loss of *TP53* can activate a dedifferentiation and EMT program [7]. Vice versa, *GNAS* mutations strengthen the IPMN-PDAC sequence [8, 9]. Other mutations have been shown to display context-dependent effects depending on their cell type-specific loss in the pancreas. In this way, PTEN fosters an IPMN-PDAC sequence together with oncogenic KRAS when selectively being removed from ducts [10], while the acinar depletion accelerates an alternative PanIN-PDAC program [11]. RNF43 is frequently mutated in intraductal papillary mucinous neoplasm (IPMN) and mucinous cystic neoplasm [12] and confers WNT-dependent growth in PDAC [12]. Therefore, PTEN and RNF43 are highly relevant for IPMN progression, likely KRAS dependent, and thereby can promote an alternative route towards PDAC. Anyhow, this is not taken into account for today's standard of care in PDAC which disregards this heterogeneity by use of conventional chemotherapies [13, 14] and thereby being far from personalized [3]. More detailed and facilitated modeling of PDAC biology respecting the subtypes with their mutational spectrum would help for better understanding and the development of new therapeutic treatments [15]. But common standard models used in pancreatic cancer research face diverse problems. Indeed, 2D cancer cell line cultures lack tumor heterogeneity and tend to accumulate mutations over time. Genetically engineered mouse models allow to overcome these problems and were fundamental in deciphering relevant biological processes in PDAC, but are limited due to their nonhuman background [16]. More closely are patient-derived tumor xenografts (PDX), where freshly resected PDAC pieces are directly transplanted into immunocompromised mice and therefore can reflect the *in vivo* situation in view of tumor heterogeneity [17, 18]. In conclusion, we have several well-established models available that can recapitulate various aspects of human PDAC evolution. However, most of the models are highly labor intensive by means of establishment, time and capacity and are not always suitable for high throughput drug screens. A promising model to overcome these evident problems are pancreatic organoids. Generally, organoids are three-dimensional (3D) model systems that highly precise reflect *in vivo* architecture and multilineage differentiation of certain tissues [19]. Organoids are based on pluripotent or organ-specific stem cells that are processed and grown under selective conditions on Matrigel [19]. Here, we generated pancreatic ductal organoids from mice [20, 21]. Genetic engineering was feasible and affected the appearance of the organoids. In summary, our data underlines the potential of organoids as a role model for different routes of PDAC evolution, like IPMN derived.

## 2. Materials and Methods

### 2.1. Ethics Statement

All animal care and procedures followed German legal regulations and were previously approved by the governmental review board of the state of Baden-Württemberg. All aspects of mouse work were carried out following strict guidelines to insure careful, consistent, and ethical handling of the mice.

### 2.2. Mouse Strains

Wild-type murine organoids were isolated from C57BL6/J mice obtained from the animal facility of Ulm University. The KC (LSL-KrasG12D/+, Ptf1a-Cre+/-) mouse was obtained by crossing LSL-KRAS^G12D^ (B6;129S4-KrasTm4Tyj/J) and *Ptf1a-Cre* (B6;129-Ptf1atm1.1(CRE)Cvw) mice.

### 2.3. Isolation and Culture of Ductal Organoids

Immediately after isolation of the murine pancreas, the tissue was minced into 0.5-1 mm fragments and digested with collagenase/dispase (Roche, 11097113001) for 30 min at 37°C and then with accutase (Sigma-Aldrich, A6964) for 30 min at 37°C. The cells were then filtered in 40 *μ*M EASYstrainer (Greiner bio-one) and put in culture on Matrigel growth factor reduced (Corning) coated plates with organoid culture medium containing 5% growth factor reduced (GFR) Matrigel (Discovery Labware, 354230). The organoid culture medium (PDC) was the one published by Reichert et al. [21]. Medium was changed every third day.

### 2.4. Lentivirus Production and Infection

Lentiviruses containing validated sh*Rnf43* (TRCN0000040790) and sh*Pten* (TRCN0000322421) were purchased from Sigma-Aldrich. The lentiviruses were produced using PEI (Polysciences, 23966) transfection, the plasmids psPAX2 (Addgene #12260) and pMD2.G (Addgene #12259), and Lenti-X cells (Clontech). For each infection, 10.000 single cells were resuspended in 1 ml PDC medium. Polybrene was added with a final concentration of 8 *μ*g/ml. Cell/polybrene/virus mix was centrifuged for 20 min at 800 rpm at RT. The pellet was resuspended in 100 *μ*l PDC medium containing 5% Matrigel and plated in a Matrigel-GFR coated well of a 96-well plate. Selection started after 24 h incubation at 37°C by changing the medium to PDC medium containing 5% Matrigel supplemented with 3 *μ*g/ml puromycin.

### 2.5. Cloning of the KRAS G12D-pLIX-403 Plasmid

Cloning steps included excision of an insert containing KRAS^G12D^ (576 bp) from the pBabe-KRAS^G12D^ plasmid (Addgene #58902) using the restriction enzymes BamHI HF (NEB) and Sal-I (NEB). Subcloning followed into pBLSK II+/- (Stratagene, KRAS^G12D^-pBLSK). The gateway KRAS^G12D^-pENTR1A plasmid was created by cutting the KRAS^G12D^ insert from KRAS^G12D^-pBLSK using the restriction enzymes BamHI HF (NEB) and Xho-I (NEB) followed by subcloning into pENTR1A (Invitrogen #A10462). Finally, the cloning of the KRAS^G12D^ insert into pLIX-403 (Addgene #41395) was performed using the Gateway technology (Invitrogen).

### 2.6. Semiquantitative RT-PCR

Total RNAs were extracted using the RNeasy Mini Kit (Qiagen). First-strand cDNAs were prepared using 250 ng of RNA and SuperScript II Reverse Transcriptase in the presence of random primers (Thermo Fisher Scientific) according to the manufacturer's protocol. Quantitative PCR were performed using an Applied Biosystems QuantStudio 3 System (annealing temperature 60°C) and PowerUp SYBR Green Master Mix (Thermo Fisher Scientific). All the real-time values were averaged and compared using the threshold cycle (CT) method, where the amount of target RNA (2-ΔΔCT) was normalized to the endogenous expression of 18S (18S ribosomal RNA) (ΔCT). The amount of target mRNA in control cells was set as the calibrator at 1.0. The following primers used for quantitative RT-PCR were purchased from Biomers, Sigma-Aldrich, or Qiagen: *Hmbs* (Qiagen, QT00494130); *Gapdh* (Qiagen, QT01658692); *KRAS* (Qiagen QT00083622); *Rnf43* (Biomers, forward 5′-gcgggtctggagaaagctac-3′, reverse 5′-agttgaccaccgagtcactg-3′); *Pten* (Biomers, forward 5′-acagccatcatcaaagagatcgt-3′, reverse 5′-tgttcctgtatacaccttcaagtct-3′); *Amylase* (Thermo Fisher Scientific, forward 5′-tggcgtcaaatcaggaacatg-3′, reverse 5′-aaagtggctgacaaagcccag-3′); *Sox9* (Sigma-Aldrich, forward 5′-aggaagctggcagaccagta-3′, reverse 5′-tccacgaagggtctcttctc-3′); *Ck19* (Biomers, forward 5′-agggccttgagattgagctg-3′, reverse 5′-tgggcttcaaaaccgctgat-3′); *Muc2* (Biomers, forward 5′-gaagccagatcccgaaacca-3′, reverse 5′-gcttcaggtgcacagcaaat-3′); and *Pdx1* (Biomers, forward 5′-ctccctttcccgtggatgaa-3′, reverse 5′-taggcagtacgggtcctctt-3′).

### 2.7. Immunostaining and Antibodies

For immunofluorescence of the ductal organoids, 10.000 single cells were seeded in an 8 Chamber Well Slide coated with Matrigel-GFR (LAB-TEK, #440263 0903). After 5 days, the organoids were washed twice with PBS 1X and fixed with 2% buffered paraformaldehyde for 20 min at room temperature. Subsequently, the organoids were again washed 3 times with PBS 1X and then permeabilized with Triton 0.7% for 15 min at RT. After blocking for 1 hour at RT (normal goat serum 5%, BSA 1%, Triton 0.4%), the primary antibodies were incubated overnight at 4°C. The following antibodies were used: cytokeratin 19 (TROMA-III, DSHB), Ki-67 (MA5-14520, Invitrogen), SOX9 (AB5535, Millipore), FOXA2 (Ab108422, Abcam), and PDX1 (AF2419, R&D). Images were acquired on an Axioplan2 microscope (Carl Zeiss) equipped with an AxioCamHR camera and AxioVision Version 4.8 (both from Carl Zeiss) software. Magnifications are given in figure legends.

### 2.8. Organoid Formation Assay

Organoids were dissociated into single cells. 5000 single cells were seeded in a Matrigel-GFR-coated well of a 24-well plate. Pictures were taken 8 days after the seeding (12 pictures with 40x magnification per well). The number and size were assessed by ImageJ. All cell viability experiments were conducted in triplicate.

### 2.9. Statistical Analysis

For statistical analysis, two-tailed Student's *t*-test was used. A *p* value of <0.05 was considered to be statistically significant. Graphs are presented with the standard error of the mean (SEM). GraphPad Prism 7 was used for statistical analysis and graphical presentation.

## 3. Results

### 3.1. Mouse-Developed Pancreatic Organoids Reflect Ductal Differentiation and Can Be Genetically Engineered

**Figure 1:**
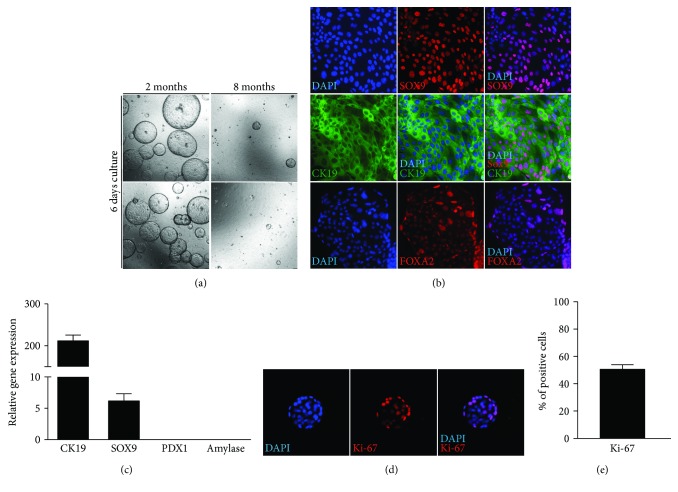
Pancreas mouse organoids exhibit ductal origin. (a) Organoid size and numbers are depending on the age of the host, with decreased numbers and size when generated from eight compared to two months old WT mice. Organoids shown are low passages 5-7 (40x magnification). (b) Organoids are positive for CK19, FOXA2, and SOX9 (200x magnification). (c) Relative gene expression levels of ductal markers (CK19 and SOX9) and lack of expression of acinar and islet cell markers amylase and PDX1. Gene expression is normalized to HMBS. (d) Ki-67 immunofluorescence (200x magnification). (e) 49.7% of the cells are positive for Ki-67 in WT organoids.

Adaptation and simplification of already published protocols [20–22] allowed us to isolate without cumbersome steps such as DBA-positive cell sorting [22] to successfully propagate ductal pancreatic organoids from 2 months old mice. Interestingly, the growth capacity of organoids significantly reduced with the age of the mice (Figure 1(a)). To confirm the ductal origin of the organoids, the state-of-the-art immunohistochemistry of ductal markers SOX9, CK19, and FOXA2 was performed. CK19 and FOXA2 were ubiquitously expressed in the organoids in which the vast majority of the cells were positive, while SOX9 was expressed in a smaller fraction of the cells (Figure 1(b)). Immunochemistry data were confirmed by RNA expression (Figure 1(c)). Indeed, CK19 and to a lesser extent SOX9 were highly expressed in the organoids while PDX1 and amylase as acinar and islet cell counterparts were not expressed at all (Figure 1(c)). Moreover, the ductal organoids were highly proliferative with around 50% of the cells Ki-67-positives (Figures 1(d) and 1(e)).

### 3.2. *Pten* and *Rnf43* Loss Supports Ductal Features in Wild-Type Organoid Cultures

**Figure 2:**
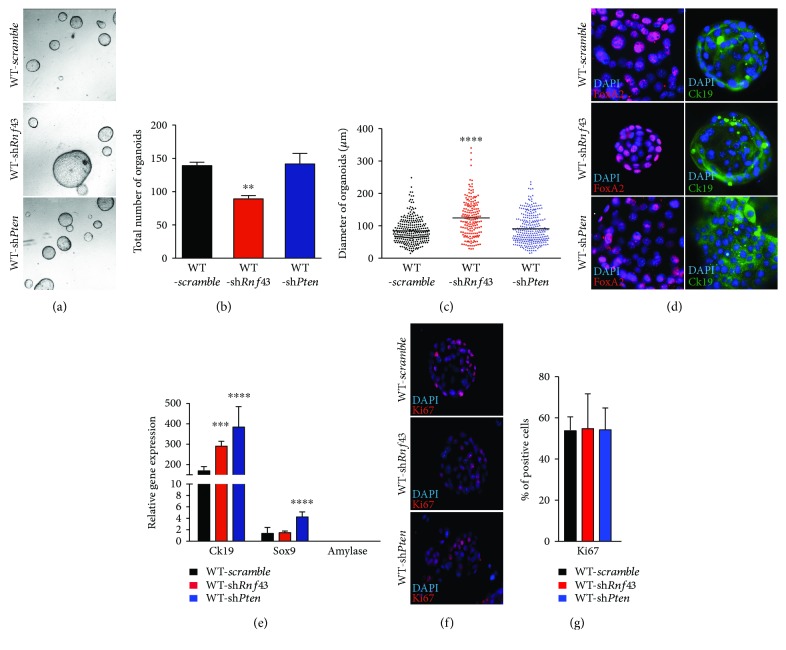
*Pten* and *Rnf43* loss supports ductal features in wild-type organoid cultures. (a) Representative pictures of organoids with shRNA knockdown of *Pten* (WT-sh*Pten*) or *Rnf43* (WT-sh*Rnf43*) compared to WT scramble control after six days of culture (40x magnification). (b) Significantly decreased organoid growth rate after knockdown of *Rnf43* (*p* = 0.0071). (c) RNF43 deficiency significantly increases the diameter of ductal organoids compared to WT scramble (*p* ≤ 0.0001). (d) Immunofluorescence stainings of shRNA-mediated knockdown *Pten* and *Rnf43* organoids reveal the expression of FOXA2 and CK19 (400x magnification). (e) The knockdown of *Pten* (WT-sh*Pten* blue bar) significantly increases the expression of CK19 (*p* ≤ 0.0001) and SOX9 (*p* ≤ 0.0001) and for CK19 (*p* = 0.001) in case of *Rnf43* (WT-sh*Rnf43* red bar) knockdown compared to WT scramble (black bar). (f) Ki-67 immunostaining analyses of shRNA-mediated knockdown of *Rnf43* and *Pten* in WT organoids (400x magnification). (g) Immunohistochemistry revealed no differences in the percentage of Ki-67-positive cells in WT-sh*Pten* (blue bar) or WT-sh*Rnf43* (red bar) compared to the scramble control (black bar). For statistical analysis, two-tailed Student's *t*-test was used. *p* < 0.05 was considered to be statistically significant. Error bars represent the standard errors of the mean.

Next, we wanted to challenge these ductal organoid cultures by removing tumorigenic roadblocks known to confer cystic growth in the context of ductal origin specifically PTEN and RNF43 [11, 12]. To establish conditions allowing the genetic modification of ductal organoids, robust shRNA knockdown of *Pten* and *Rnf43* was performed (Suppl Fig.  1A-B). The knockdown of *Pten* did not show any numerical or morphologic changes compared to WT organoids (Figures 2(a)–2(c)). In contrast, the knockdown of *Rnf43* significantly impaired the self-renewal (*p* = 0.0071) and vice versa resulted in enlarged organoids (*p* = 0.0001) (Figures 2(a)–2(c)). Expression levels of ductal markers CK19 and SOX9 were significantly elevated in response to PTEN deficiency, while RNF43 loss just upregulated CK19 (Figures 2(d) and 2(e)). Surprisingly, the increased size of organoids after *Rnf43* knockdown did not affect the proliferation rate suggesting that the increased size is rather associated with structural cell architecture modification and organoid swelling (Figures 2(f) and 2(g)).

### 3.3. *Pten* and *Rnf43* Loss Cooperates with Kras to Foster Oncogenic Growth in Ductal Organoids

**Figure 3:**
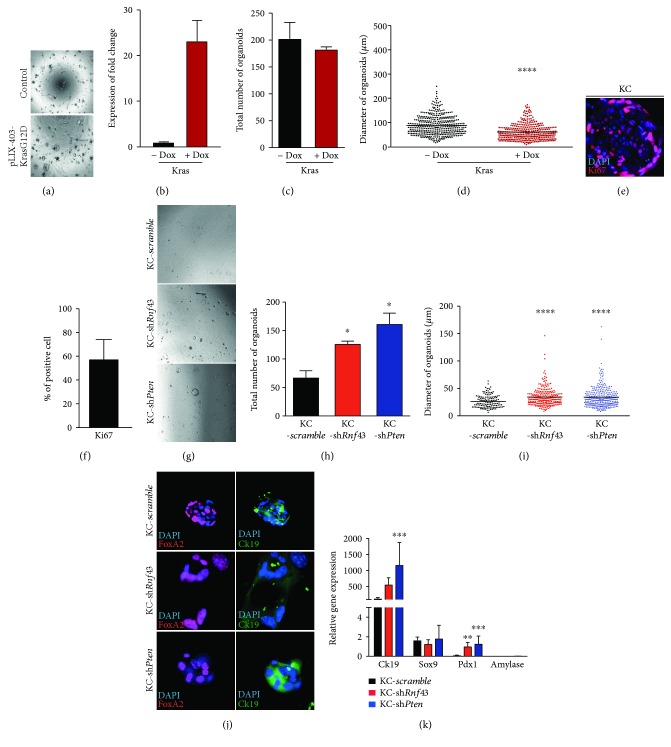
Oncogenic Kras fosters proliferation in PTEN- and RNF43-deficient ductal organoids. (a) Doxycycline-inducible KRAS^G12D^ (pLIX-403-KRAS^G12D^) and control organoids are morphologically similar after six days of culture (40x magnification). (b) KRAS^G12D^ expression can be induced by doxycycline application in the pLIX-403-KRAS^G12D^ organoids. (c, d) KRAS^G12D^ induction does not change the number of organoids but significantly the size compared to control (*p* ≤ 0.0001). (e, f) In total, 57.6% of the cells are positive for Ki-67 (400x magnification). (g) Representative pictures of KC organoids with shRNA knockdown of *Pten* (KC-sh*Pten*) or *Rnf43* (KC-sh*Rnf43*) compared to KC scramble control after eight days of culture (40x magnification). (h) Significant differences are observed in the number of organoids for KC-sh*Pten* (*p* = 0.0276) and KC-sh*Rnf43* (*p* = 0.0234) organoids compared to control. (i) The diameter of KC organoids is significantly increased after knockdown of *Pten* (*p* < 0.0001) and *Rnf43* (*p* < 0.0001). (j) Immunofluorescence staining of FOXA2 and CK19 in KC organoids with either shRNA-mediated knockdown of *Pten* (KC-sh*Pten*) or *Rnf43* (KC-sh*Rnf43*) (400x magnification). (k) Relative gene expression levels are significantly elevated for CK19 (*p* = 0.0004) and PDX1 (*p* = 0.0006) in KC-sh*Pten* (blue bar) and for PDX1 (*p* = 0.037) in KC-sh*Rnf43* (red bar) compared to KC scramble (black bar). Amylase is not expressed. For statistical analysis, two-tailed Student's *t*-test was used. *p* < 0.05 was considered to be statistically significant. Error bars represent the standard errors of the mean.

To display the net effect of oncogenic Kras in ductal organoids, we generated a doxycycline-inducible KRAS^G12D^ overexpressing line (Figures 3(a) and 3(b)). No numerical but differences in the size could be seen between WT organoids and upon KRAS^G12D^ induction (Figures 3(c) and 3(d)). Doxycycline treatment itself also seemed to have a minor effect on the growth of WT organoids that however was significantly less than the effect of Kras activation (Suppl Fig.  1C-D). To further correlate our results with a genetically better defined system, we isolated ductal cells from a *Kras^G12D/+^ Ptf1a^Cre/+^* mouse (KC) (Figures 3(e) and 3(g)). KC organoids appeared slightly bigger compared to WT organoids and were more proliferative as shown by Ki-67 staining (Figure 3(f)). As oncogenic *KRAS* is known as a major event in the IPMN-PDAC sequence, we further evaluated *Pten* and *Rnf43* depletion in the context of constitutively overexpressed KRAS^G12D^ (KC) (Figure 3(g)) using the above described lentiviral knockdown system on KC ductal organoids. To our surprise, oncogenic KRAS^G12D^ in concert dramatically changed the organoid characteristics upon PTEN and RNF43 loss. Specifically, this forced a significant increase of self-renewal in KC-sh*Pten* (*p* = 0.0276) and KC-sh*Rnf43* (*p* = 0.0234) organoids compared to KC scramble control (Figures 3(h) and 3(i)). Next, we wanted to know whether the cystic growth per se of the pancreatic ductal organoids is altered in light of both oncogenes. While in the absence of oncogenic KRAS solely RNF43 loss increased cyst size, its presence ascribed in both RNF43 and PTEN knockdown organoids a more cystic growth pattern compared to scramble controls. Finally, we wanted to know again whether the previously shown ductal marker upregulation can be potentiated by oncogenic KRAS. Indeed, significant differences were again observed in the expression of CK19 (*p* = 0.0004) referring to PTEN and RNF43-deficient (*p* = 0.037) KC organoids (Figures 3(j) and 3(k)). Oncogenic growth in PDAC is linked to the activation of embryonic programs and dedifferentiation phenotypes [23]. Thus, we assessed again the normally low expressed progenitor marker PDX1 in various organoid types. Indeed, KRAS^G12D^ overexpression combined with loss of either RNF43 or PTEN increased PDX1 expression (Figure 3(k)). These results indicate that the sole depletion of the tumor suppressor genes *Rnf43* and *Pten* is not sufficient to trigger proliferation and self-renewal capacity, but activation of an additional protooncogene is required.

## 4. Discussion

Within the present paper, we could establish and genetically engineer murine pancreatic ductal organoids. Optimization of the isolation and culture conditions was achieved by combining and adapting published protocols [20, 21]. The current protocol allows isolation in a short time of ductal organoids with a success rate close to one hundred percent. Organoids were able to be passaged up to thirty times without losing proliferation capacity and morphology. The ductal origin could be clearly verified by expression of established markers like SOX9, CK19, and FOXA2 on the one hand. On the other hand, PDX1 and amylase markers for acinar and islet cell differentiation remained almost negative and thereby underline the pure ductal phenotype. In the matured pancreas, the expression of PDX1 is restricted to insulin-producing *β*-cells [24], but is also found to be upregulated in pancreatic cancer [25]. Our data for PDX1, SOX9, and CK19 expression is in line with the pancreatic organoid model from Boj et al. [19]. However, it is conflicting referring to Broutier et al.'s showing higher expression of PDX1 [22]. The discrepancies might be explained by a selective isolation of PDX1-negative cells using our protocol or by means of age, albeit the expression of PDX1 is increasingly compartment specific throughout pancreas maturation.

Consequently, the organoids were genetically engineered by lentiviral shRNA transduction for depletion of the tumor suppressor genes *Pten*, *Rnf43*, and cDNA to overexpress KRAS^G12D^. The use of lentiviruses allowed an easy approach to verify the value of the organoid system in the light of well-characterized genes in IPMN and PDAC formation. To our knowledge, this is the first attempt to model these specific mutations in mouse pancreatic organoids. To diminish lentiviral promoted side effects, such as random or multiple copy integration, alternatively, one could consider the use of CRISPR/Cas9 systems for future research. The overexpression of KRAS^G12D^ was directed constitutively or in a doxycycline-dependent manner. A pitfall of doxycycline use is a potential interference that has already been shown at low doses on highly sensitive cells such as neurons [26]. In fact, our model showed mild impairment after doxycycline exposition. The sole KRAS^G12D^ overexpression fostered proliferation in KC organoids, but did not affect *in vitro* growth of the organoids.

Oncogenic KRAS^G12D^ is a major event in PDAC development and induces a slow formation of PanIN lesions *in vivo* [27], though accumulation of further somatic mutations like *TP53* [28], *TGFb receptor type 2* (TGFBR2) [29], or *SMAD4* [30] is warranted to accelerate PDAC formation. Vice versa, the absence of oncogenic *Kras* limited the development of PDAC. To evaluate our organoid model in this scenario, we evaluated the knockdown of well-known and relevant PDAC tumor suppressor genes that were not transferred to mouse ductal organoids by now. Thereby, the selective knockdown of *Pten* showed no obvious effect on proliferation or self-renewal capacity of the ductal organoids. In the context of oncogenic *Kras*, self-renewal and proliferation capacity were significantly increased. Appropriately, synergistic functions of the PTEN/PI3K/AKT and RAS/RAF/MAPK pathways are described in promoting pancreatic cancer initiation and progression, partially by strong activation of the NF-KB network [11, 31]. The knockdown of *Rnf43* on its own already influenced the proliferation and self-renewal capacity in our organoids and was further accelerated by oncogenic KRAS*^G12D^*. Generally, the ubiquitin E3 ligase Rnf43 is a negative feedback regulator of Wnt signaling by degradation of Frizzled that could be inhibited by FZD5 antibodies [32, 33]. In line with our findings, *Rnf43* inactivation is published to promote cytosolic *β*-catenin, Wnt/*β*-catenin signaling, and thereby proliferation [12]. Moreover, we could show that activation of oncogenic programs in our organoids (KC-shPten and KC-shRnf43) came along with increased PDX1 expression as a surrogate for restored embryonic programs and dedifferentiation.

The aforementioned selectively applied mutations in our pancreatic ductal organoids basically reproduced effects published *in vivo* in the literature and thereby fulfilled the “proof of concept” approach.

In summary, the data presented underlines the potential of organoids as a role model for PDAC evolution and forms the basis of further applications like high throughput drug screens.

## Data Availability

The data used to support the findings of this study are included within the article.

## Supplementary Materials

Supplementary Materials Supplementary Figure 1: (A) Proof of knockdown for *Rnf43* and *Pten* by expression fold change referred to WT organoids. (B) Organoid culture for WT scramble and knockdown of *Pten* and *Rnf43* reveal no differences after 6 days of culture. (40x magnification). (C) Organoid self-renewal assay of WT organoids upon doxycyclin treatment showing no significant difference between nontreated (-Dox) and treated (+Dox) WT organoids. (D) doxycyclin treatment impairs WT organoid growth (*p* = 0.0028). Kras activation significantly reduces organoid growth compared to WT organoids independent of Dox treatment (*p* < 0.0001). For statistical analysis, two-tailed Student's *t*-test was used. *p* < 0.05 was considered to be statistically significant. Error bars represent standard errors of the mean.
