# Supplementary figures and images for: Pancreatic Ductal Organoids React Kras Dependent to the Removal of Tumor Suppressive Roadblocks
Source: Stem Cells Int. 2019 May 19;2019:2079742. doi: 10.1155/2019/2079742 (PMC6545725; doi:10.1155/2019/2079742)

A

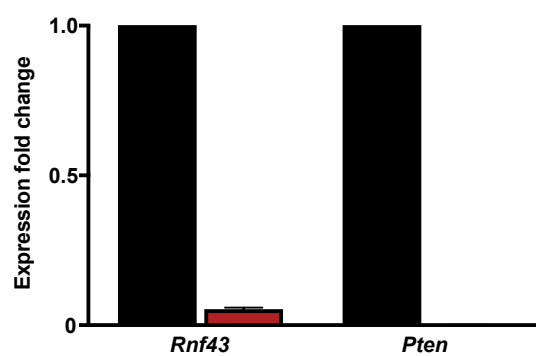

B

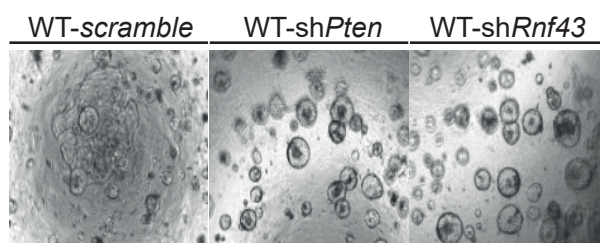

C

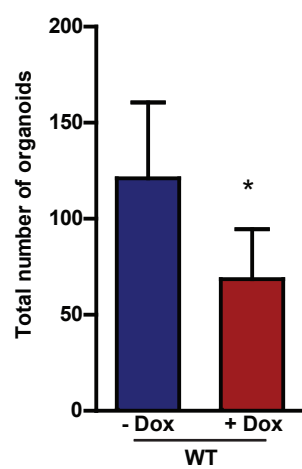

D

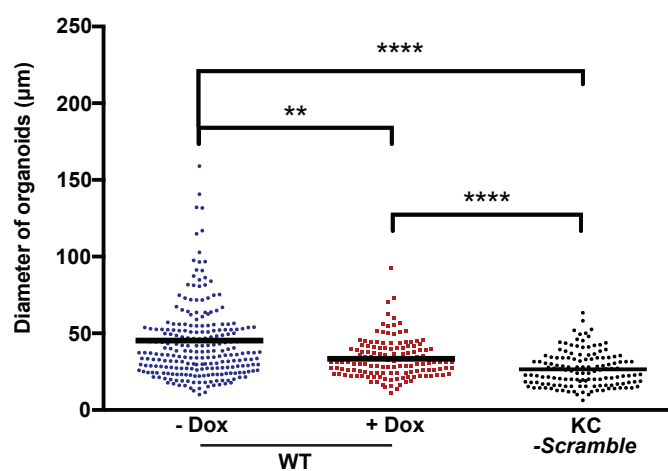

Supplementary Figure 1

Supplement: Supplementary Materials — Supplementary Figure 1: (A) Proof of knockdown for Rnf43 and Pten by expression fold change referred to WT organoids. (B) Organoid culture for WT scramble and knockdown of Pten and Rnf43 reveal no differences after 6 days of culture. (40x magnification). (C) Organoid self-renewal assay of WT organoids upon doxycyclin treatment showing no significant difference between nontreated (-Dox) and treated (+Dox) WT organoids. (D) doxycyclin treatment impairs WT organoid growth (p = 0.0028). Kras activation significantly reduces organoid growth compared to WT organoids independent of Dox treatment (p < 0.0001). For statistical analysis, two-tailed Student's t-test was used. p < 0.05 was considered to be statistically significant. Error bars represent standard errors of the mean. [file 2079742.f1.pdf]
